# Intrabodies targeting human papillomavirus 16 E6 and E7 oncoproteins for therapy of established HPV-associated tumors

**DOI:** 10.1186/s13046-021-01841-w

**Published:** 2021-01-23

**Authors:** Francesca Paolini, Carla Amici, Mariantonia Carosi, Claudia Bonomo, Paola Di Bonito, Aldo Venuti, Luisa Accardi

**Affiliations:** 1 HPV Unit, UOSD Tumor Immunology and Immunotherapy, IRCCS Regina Elena National Cancer Institute, 00144 Rome, Italy; 2 Department of Biology, University of Rome Tor Vergata, 00133 Rome, Italy; 3 Anatomy Pathology Unit, Department of Research, Diagnosis and Innovative Technologies, IRCCS-Regina Elena National Cancer Institute, 00144 Rome, Italy; 4 Department of Infectious Diseases, Istituto Superiore di Sanità, 00161 Rome, Italy

**Keywords:** HPV-associated cancer, Antitumor intracellular antibodies, Therapeutic single-chain antibody fragments, HPV16 E6 and E7 oncoproteins, Apoptosis, Gene delivery by electroporation

## Abstract

**Background:**

The oncogenic activity of the high risk human papillomavirus type 16 (HPV16) is fully dependent on the E6 and E7 viral oncoproteins produced during viral infection. The oncoproteins interfere with cellular homeostasis by promoting proliferation, inhibiting apoptosis and blocking epithelial differentiation, driving the infected cells towards neoplastic progression. The causal relationship between expression of E6/E7 and cellular transformation allows inhibiting the oncogenic process by hindering the activity of the two oncoproteins. We previously developed and characterized some antibodies in single-chain format (scFvs) against the HPV16 E6 and E7 proteins, and demonstrated both in vitro and in vivo their antitumor activity consisting of protective efficacy against tumor progression of HPV16-positive cells.

**Methods:**

Envisioning clinical application of the best characterized anti-HPV16 E6 and –HPV16 E7 scFvs, we verified their activity in the therapeutic setting, on already implanted tumors. Recombinant plasmids expressing the anti-HPV16 E6 scFvI7 with nuclear targeting sequence, or the anti-HPV16 E7 scFv43M2 with endoplasmic reticulum targeting sequence were delivered by injection followed by electroporation to three different preclinical models using C57/BL6 mice, and their effect on tumor growth was investigated. In the first model, the HPV16+ TC-1 Luc cells were used to implant tumors in mice, and tumor growth was measured by luciferase activity; in the second model, a fourfold number of TC-1 cells was used to obtain more aggressively growing tumors; in the third model, the HPV16+ C3 cells where used to rise tumors in mice. To highlight the scFv possible mechanism of action, H&E and caspase-3 staining of tumor section were performed.

**Results:**

We showed that both the anti-HPV16 E6 and HPV16 E7 scFvs tested were efficacious in delaying tumor progression in the three experimental models and that their antitumor activity seems to rely on driving tumor cells towards the apoptotic pathway.

**Conclusion:**

Based on our study, two scFvs have been identified that could represent a safe and effective treatment for the therapy of HPV16-associated lesions. The mechanism underlying the scFv effectiveness appears to be leading cells towards death by apoptosis. Furthermore, the validity of electroporation, a methodology allowed for human treatment, to deliver scFvs to tumors was confirmed.

**Supplementary Information:**

The online version contains supplementary material available at 10.1186/s13046-021-01841-w.

## Background

Papillomaviruses were the first class of viruses to be associated with human cancer [1]. Out of over 200 Human Papillomaviruses (HPV) genotypes, only twelve to fourteen, defined as high risk (HR) types, are etiologically involved in virtually all squamous cell carcinomas (SCC) of the cervix, a high percentage of those in the ano-genital area and an increasing fraction of the head and neck cancers (HNSCC) [2, 3].

The causal relation between HPV infection and cancer allowed the development of prophylactic vaccines able to prevent cancer but not intended to cure preexisting infections [4]. Therefore, HR HPV genotypes can persist unapparent after infection and cause the onset of lesions and progression to cancer over years or decades, reliant on co-factors [5].

The HPV oncogenicity is primarily dependent on the continuous expression and activity of the E6 and E7 viral proteins, which are Tumor-associated antigens (TAAs) acting in concert to alter interrelated cellular processes and promoting tumor development through the interaction with over 100 different cellular proteins [6]. E6 and E7 are also recognized as tumor rejection antigens thus representing valid targets for therapeutic vaccination. Hence, two kinds of therapeutic approaches targeting the E6 and E7 oncoproteins are usually implemented. The first one is based on stimulation of the cell-mediated immune response arming E6- and E7-specific CTLs able of rejecting the HPV tumor [7]. The second one is based on the direct blocking of their oncogenic activity through specific monoclonal antibodies (mAbs) [8].

Therapeutic drugs based on mAbs are largely represented in the biotechnology industry, whereby the European Medicines Agency and the US Food and Drug Administration have approved ninety-eight antibody therapies for the European or US market up to date and sixteen are under review (Antibody Society. Approved antibodies. Available at https://www.antibodysociety.org/resources/approved-antibodies/) [9]. In this context, the antibodies in single chain format (scFvs) are well represented due to characteristics which make them suitable to multiple purposes, such as the capacity of effectively inhibiting different protein functions demonstrated by several anticancer applications both in vitro and in vivo [10, 11].

The little-sized scFv format allows ease of manipulation as it comprises only the variable domains of the heavy (VH) and light (VL) Immunoglobulin chains, joined by a flexible linker. The scFv molecules can be engineered according to the purpose, e. g. by grafting the Complementarity Determining Regions (CDR) into different plasmid scaffolds for expression in prokaryotic cells for purification as proteins, or in eukaryotic cells, by viral or non-viral vectors, even as intracellular antibodies (intrabodies) targeting intracellular harmful molecules [12, 13].

It is notable that most therapeutic mAbs either in single-chain or classical format approved so far or under investigation for anti-cancer purposes, target proteins localized on the surface of transformed or infected cells. Instead, in the HPV system, the localization of oncoproteins only in the infected cells prompted us to develop the potential therapeutic scFvs against the oncoproteins as intrabodies.

We previously characterized several scFvs specific for the E7 and E6 oncoproteins of HPV16 (16E7 and 16E6, respectively) in terms of binding epitopes and biophysical features. Two of them were expressed as intrabodies in HPV16-positive tumor cells and showed antitumor activity: the anti-16E7 scFv43M2SD with signal for localization in endoplasmic reticulum (ER) and the anti-16E6 scFvI7nuc with signal for nuclear localization (NLS). Such scFvs were able to hamper cell proliferation and favor apoptosis in vitro in cellular systems, and hindered or delayed neoplastic growth in animal models, in preventive setting [14–16].

In this study, we implement data supporting an effective use of scFv43M2 and scFvI7nuc in tumor therapy. For this purpose, we investigated the antitumor effect of scFvs delivered as intrabodies by electroporation (EP) to HPV16-positive tumors implanted in mice. In addition to being an effective in vitro gene transfer method, EP is emerging as a method for delivery of chemotherapeutics to human tissues [17]. Since a typical feature of HPV cancer is the growth in well-defined areas, EP could represent a delivery system applicable to the treatment of HPV lesions in humans.

The results, obtained with three different HPV tumor models, confirmed the ability of anti-16E6 and –16E7 scFvs to induce a marked inhibition of tumor growth. Importantly, the anti-tumor treatment was associated with the presence of large apoptotic areas in tumors, substantiating the hypothesis that the scFv-induced perturbation of the E6 or E7 activity can trigger cell death pathways in HPV16+ tumors as already reported in vitro in HPV16+ cells [16].

## Methods

### ScFv constructs and cell lines

Selection of the anti-16E7 scFv43 from the ETH-2 library and scFvI7 from the SPLINT library was previously described [14, 16]. ScFv43 was subjected to site-directed mutagenesis to improve stability and the new antibody used thereafter was named scFv43M2 [18]. By subcloning, the scFvs were provided with signals for intracellular localization: scFv43M2 was provided with SEKDEL for retention in ER, and scFvI7 with NLS for nuclear localization. The resulting scFvs were named scFv43M2SD and scFvI7nuc. Two recombinant plasmids expressing irrelevant anti-β-galactosidase scFvs, respectively provided with signals for nuclear targeting (scFvR4nuc) or secretory leader sequence (R4sec), provided by A. Cattaneo [19] were used as controls.

The murine TC-1 cell line, derived from primary lung epithelial cells co-transformed with the HPV16 E6-E7 and activated c-H-Ras oncogenes [20], and C3 cell line, derived from embryonic mouse cells transformed with full HPV16 genome and activated Ras oncogene [21], were grown in RPMI 1640 with 10 mmol/L of HEPES, 1 mmol/L of sodium pyruvate supplemented with 2 mmol/L of nonessential amino acids and 10% FCS. Both cell lines are passages of the original clones and were routinely checked for the presence of HPV sequence and resistance to the G418 antibiotic selection (0.4 μg/ml). They are able to establish subcutaneous tumors in C57BL/6 syngeneic mice, providing models of human HPV16-associated neoplasms. TC-1-LUC cells were obtained by infection with a lentivirus containing the firefly luciferase gene that was generated according to standard procedures [22]. Cells were cultivated in the presence of 10 μg/ml Blasticidin (Merck, Italy) and those with stable LUC expression selected by luciferase assay screening.

### Therapeutic setting: intra-tumor scFv delivery in mice by electroporation

Six-eight week-old female C57BL/6 mice were obtained from Charles River Laboratories Italia, divided into four groups and maintained under specific pathogen-free conditions at the Experimental Animal Department of the Regina Elena National Cancer Institute (Rome, Italy). All experimental procedures were approved by the Institutional Animal Care of the Regina Elena National Cancer Institute and by the Government Committee of National Ministry of Health (85/2016-PR) and were carried out in accordance with EU Directive 2010/63/EU for animal experimentation. In consideration of the ethical suggestions to minimize the number of animals, 4 mice per treatment were used. Three different in vivo experiments were performed, using 5 × 10^4^ TC-1 Luc, 2 × 10^5^ TC-1 or 5 × 10^5^ C3 cells, respectively. Tumor cells were injected subcutaneously (s. c.) into the right inner flank of mice. One week after cancer cell injection, tumors were measurable and the first treatment was administered intra-tumor. Briefly, mice were anesthetized and 50 μg of scFvI7nuc or scFv43M2SD -expressing plasmids, diluted in sterile 0.9% saline solution, were injected centrally into the tumor using a 1 ml syringe with a 30-gauge needle. Recombinant plasmids expressing the irrelevant anti-β-galactosidase scFv provided with NLS (R4nuc) or secretory sequence (R4sec) were used as negative controls, respectively. Immediately after DNA injection, tumors were subjected to electroporation using a BTX ECM 830 square wave generator (Harvard Apparatus) to deliver one unidirectional pulse (100 V/cm, 50 ms) with a BTX tweezertrode array. This intra-tumor delivery setting was repeated once a week for three times, for a total of four treatments. According to the experimental plan, tumor growth was monitored once or twice a week by digital caliper measurements, or weekly by in vivo imaging. Mice were euthanized after one week from the last treatment for ethical reasons, to avoid animal suffering. In the bioluminescent approach, tumor burden was quantified by measuring the luciferase activity. Briefly, mice were anesthetized and intraperitoneal injection of 150 mg/kg of D-luciferin (Caliper, PerkinElmer, Italy) was performed. Ten minutes later, light emission was acquired for 5 min. Signal was detected using the IVIS Lumina II CCD camera system and analyzed by the Living Image 15 2.20 software package (Caliper Life Sciences, Milan, Italy). Photon emission was measured in specific regions of interest (ROI) and expressed as photon/second/cm^2^/steradian (p/s/cm^2^/sr). Higher signal intensity represents higher tumor mass. Tumor volumes were calculated by caliper measurement according to the formula V = L x W^2^ × 0.52, where V is tumor volume, L is tumor length, W is tumor width.

### Histochemical and Immunohistochemical analysis of TC-1 tumors

The presence of necrotic areas (Fig. 4) in tumor sections was evaluated by routine Hematoxylin/Eosin (H&E) staining by two independent and blinded pathologists.

For immunohistochemical staining, five μm-thick sections of paraffine-embedded mouse tumors were treated according to the procedure developed by Bonnet et al. [23]. In brief, deparaffinized and rehydrated sections on polylysine-coated glass slides were subjected to epitope retrieval in sodium citrate buffer, pH 6, for 30 min at 97 °C. After inhibition of endogenous peroxidase, the sections were incubated in humidity chamber overnight at 4 °C with rabbit anti-human/mouse active caspase-3 antigen affinity-purified polyclonal antibody (AF835; B&D Systems, Italy) diluted 1: 2000 in antibody diluent solution. Primary antibody detection and stain were performed in an automated apparatus (Leica BOND-III) with secondary biotinylated universal antibody at a dilution of 1:1000.

After the last washing, sections were H&E counterstained according to standard procedures. By this 3,3′-Diaminobenzidine (DAB)-H&E-staining (brown and blue-pink, respectively) procedure, apoptotic cells appear brown whereas necrotic cells have pink stained (eosin) nuclei and cytoplasm.

### Statistical analysis

Two-tailed Student’s t-test using the GraphPad Prism 8 software was used for correlation data. Two-way repeated measures ANOVA was applied for multiple measurements using SPSS Statistics software version 21. A *p* < 0.05 was considered statistically significant.

## Results

### Inhibition of tumor growth by anti-E6 and-E7 scFvs delivered as intrabodies

In this study, to design a treatment mimicking the therapy of already established HPV lesions in humans, we tested the anti-tumor potential of two previously characterized anti-16E6 and -16E7 scFvs in three different therapeutic settings of HPV-associated experimental tumors.

Firstly, the anti-16E6 and –16E7 scFvs ability to hamper development of TC-1 tumors was evaluated. C57/BL6 mice were inoculated s. c. in the right flank with 0.5 × 10^5^ TC-1-LUC tumor cells. After one week, when tumors were palpable, recombinant plasmids expressing scFvI7nuc or scFv43M2SD were injected intra-tumor, immediately followed by EP at the injection site. The procedure was repeated for 3 times at 1-week intervals, for a total of 4 treatments. The irrelevant anti-β-galactosidase scFvR4nuc and scFvR4sec were used as negative controls. Tumor growth was monitored weekly by measurement of luciferase activity.

**Fig. 1:**
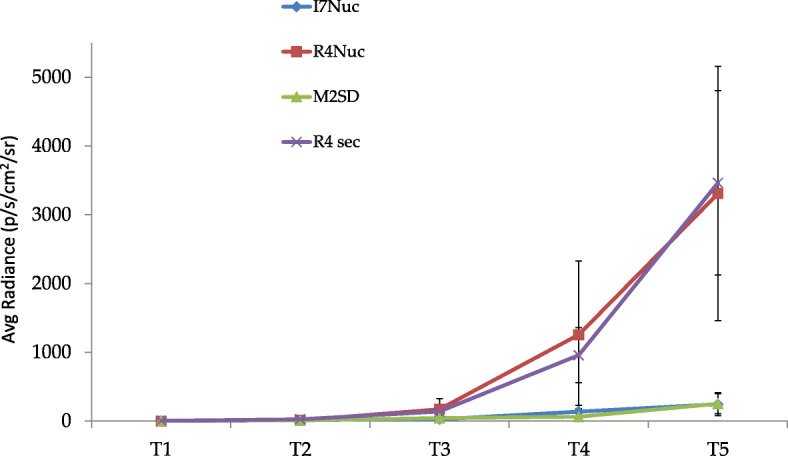
Antitumor therapeutic effect of the anti-16E6 scFvI7nuc and anti-16E7 scFv43M2SD on TC-1-Luc tumors. The graph shows the luminescent signal quantification of pooled data from tumors developed by injection of 5 × 10^4^ TC-1-LUC cells in C57/BL6 mice. The scFvI7nuc (I7nuc) and scFv43M2SD (M2SD), and the irrelevant anti-β-galactosidase scFvR4nuc (R4nuc) and scFvR4sec (R4sec) as negative controls, were delivered to tumors of randomized groups of mice, four times at one-week interval. Tumor growth was monitored with the IVIS® Lumina imaging system. Photon emission was measured in specific regions of interest (ROI) and expressed as photon/second/cm^2^/steradian (p/s/cm^2^/sr). The difference between the mean values of photon emission of therapeutic scFvs versus their controls was statistically significant (*p* = 0.0195 for I7 nuc, *p* = 0.049 for 43M2SD) as calculated at T5. T is the time point in weeks after tumor cell challenge

All mice treated with the irrelevant vectors showed progressive tumor growth. Conversely, tumor development was greatly slowed down in mice treated with scFv43M2SD or scFvI7nuc. Three weeks after tumor challenge, the difference between the luminescent signal of mice treated with the specific scFvs and the respective controls became significant. Luminescent signal quantification of pooled data from animals developing tumors are summarized in Fig. 1. A clear delay in tumor progression was evidenced upon intra-tumor treatment with anti-16E6 and -16E7 scFvs. Mice were followed for 4 weeks from tumor challenge, when the experiment was interrupted for ethical reasons to avoid animal suffering. Images of the mice treated weekly are shown in supplementary data (Fig. 1s).

**Fig. 2:**
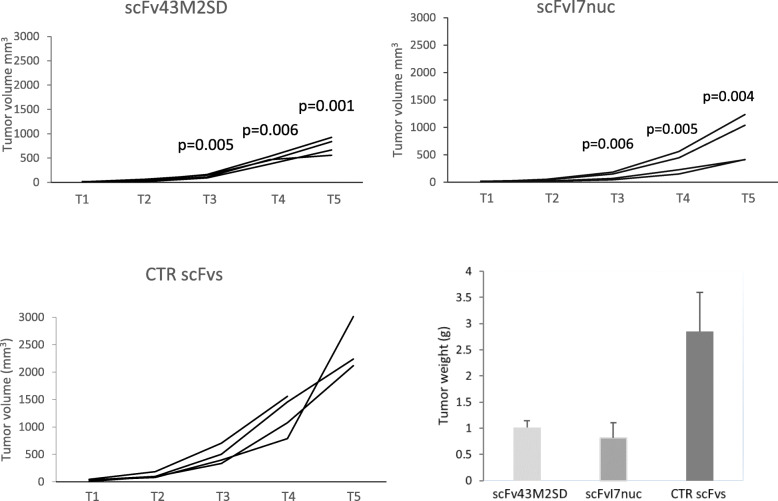
Antitumor therapeutic effect of the anti-16E6 scFvI7nuc and anti-16E7 scFv43M2SD on higher inoculum of TC-1 cells. C57/BL6 mice were injected subcutaneously with 2 × 10^5^ TC-1 cells and treated intratumor with plasmids expressing the anti-16E6 scFvI7nuc and anti-16E7 scFv43M2SD or irrelevant scFvs (CTR scFvs). Each line represents a different mouse within the same treatment group. Two types of control mice treated with R4nuc and R4sec were combined into a single group consisting of 4 total mice to keep the number of mice to a minimum. Treatment was performed four times at one-week intervals, in parallel with tumor size monitoring by caliper measurement. T is the time point in weeks after tumor cell challenge. Tumor growth is expressed as the tumor volume in mm^3^ at the indicated time points. Significant *p* values with respect to the corresponding controls are reported on the graphs. In the histogram, the mean weight ± SD of the tumors treated with therapeutic scFvs, and their controls, excised after mice sacrifice, is reported. Differences are significant with *p* = 0.004 for scFvI7nuc and *p* = 0.006 for scFv43M2SD

Secondly, in order to evaluate antitumor scFv activity in a more aggressive tumor condition, an additional experiment was performed by inoculating 2 × 10^5^ TC-1 cells s.c. in the mice right flank. One week after tumor inoculum, mice were injected intratumor with scFv43M2SD or scFvI7nuc, immediately followed by EP at the injection site. The treatment was repeated 3 times at one-week intervals, in parallel with monitoring of tumor size by caliper, which was continued for one week after the last treatment. Tumor volumes of single mice are shown in Fig. 2. Significant delay of tumor development was obtained in all mice treated with both scFvs targeting the oncoproteins but not in the control mice receiving irrelevant scFvs. In Fig. 2, the averages of tumor weight at the time of mice sacrifice are also shown. The difference between relevant scFvs and their controls confirmed efficacy and specificity of the intrabody treatment even in a more aggressive tumor setting.

**Fig. 3:**
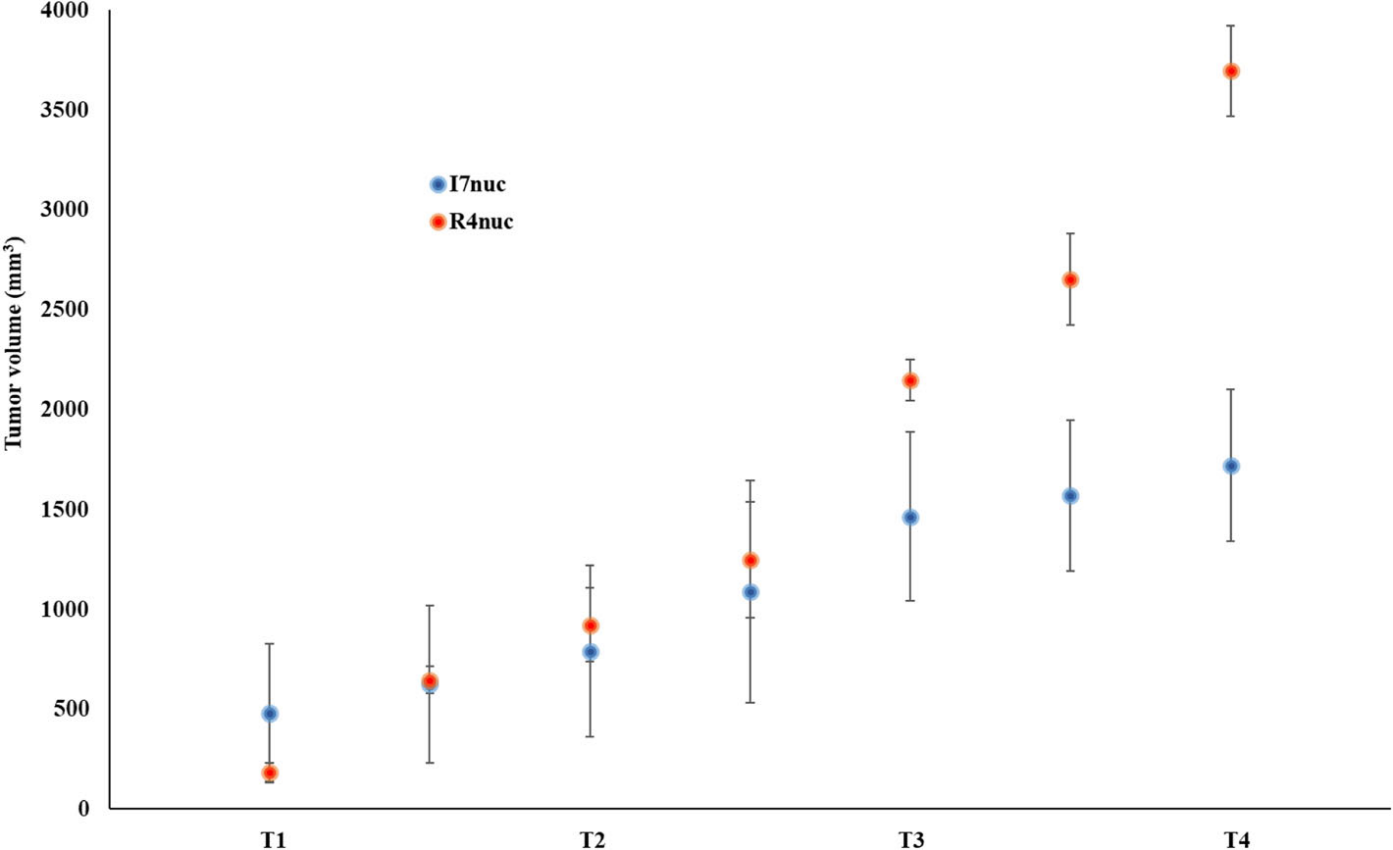
Antitumor therapeutic effect of the anti-16E6 scFvI7nuc on C3 tumors. The scatter plot shows the volume of C3 tumors raised in C57/BL6 mice treated with scFvI7nuc (I7nuc) or the unspecific scFvR4nuc (R4nuc). Each point represents the mean volume ± SD of 4 different mice, measured by caliper every 3–4 days. T is the time point in weeks after tumor cell challenge. At the end point (T4), differences are statistically significant (*p* = 0.0005) by two-tailed Student’s t-test. Two-way repeated measures ANOVA analysis showed *p* = 0.017 for treatment and *p* = 0.0001 for linear trend. Data were expressed as means ± standard deviations (SD). Of note, the tumor volume of R4nuc mice had an unexpected increase in the last 3 days prior to the measurement at T4. Despite mice were in good health, tumor burden exceeded the size allowed by our internal ethics and all mice were immediately sacrificed

Thirdly, the effect of scFvI7nuc was tested in C57/BL6 mice inoculated with 5 × 10^5^ C3 cells, which are tumor cells harbouring the full HPV16 genome, to extend applicability of the scFv therapy to a different HPV16-positive solid tumor. Similar to previous experiments, intratumor scFvs injection and electroporation in mice were started one week after tumor challenge but treatment was repeated twice at 1-week intervals. Tumor size was measured by caliper at different time intervals. As shown in Fig. 3, effective tumor growth inhibition was obtained by delivery of scFvs even in this HPV tumor model, strengthening the feasibility of such methodology for the treatment of HPV tumors. Two-way repeated measures ANOVA analysis showed high significance with *p* = 0.017 for treatment and *p* = 0.0001 for linear trend.

**Fig. 4:**
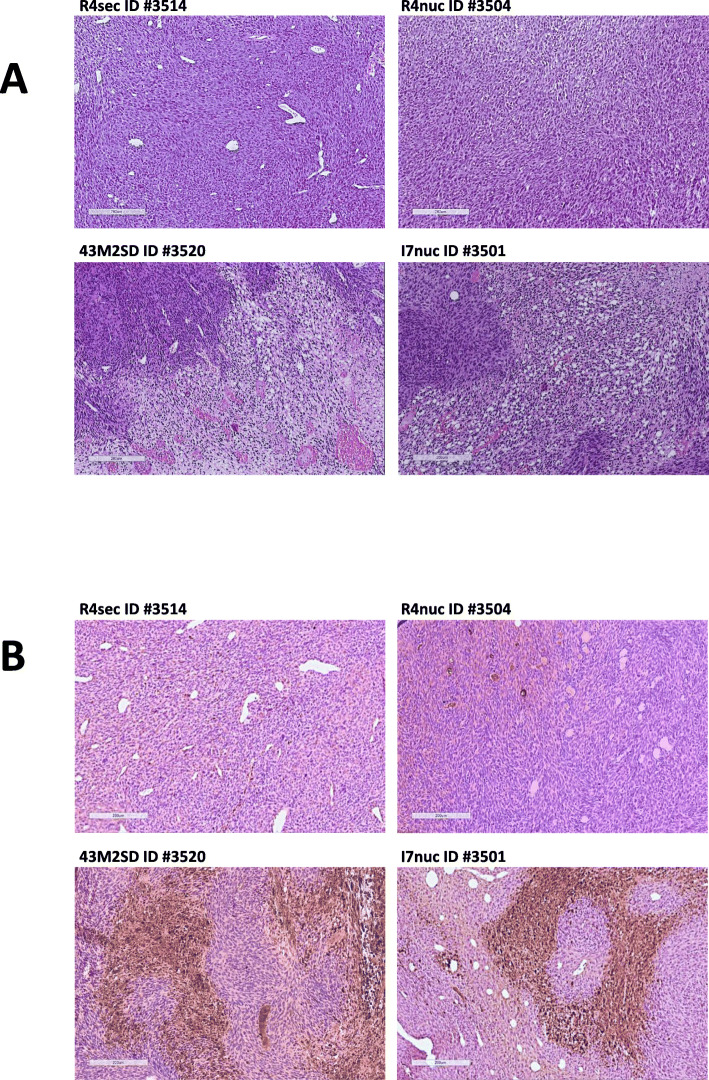
Treatment with anti-16E6 and -16E7 scFvs induces cell death by apoptosis in tumor mass. H&E staining (panels **a**) and active caspase-3 (cleaved caspase) staining (panels **b**) of HPV tumor sections from mice treated with scFvI7nuc (I7nuc), scFv43M2SD (43M2SD) and the irrelevant scFvR4nuc (R4nuc) and scFvR4sec (R4sec) are presented. Tumors from one representative mouse for each treatment group are shown. Referring to the numbering shown in Fig. s1, mouse # 3501 for I7nuc, #3504 for R4nuc, #3520 for M2SD and #3514 for R4sec were used. With H&E staining, wide areas of necrosis are visible in tumors treated with both therapeutic scFvs, while rare signs of necrosis are detected in tumors treated with the control scFvs (**a**). With active caspase-3 staining, brown-stained areas meaningful of apoptotic cell death are clearly visible in tumors treated with both therapeutic scFvs while rare apoptotic areas are detected in tumors treated with the control scFvs (**b**)

Two independent and blinded pathologists analysed tumor histology after scFv treatment. H&E staining of tumor sections showed wide areas of necrosis in the scFv-treated tumors (Fig. 4a, panels 43M2SD, I7nuc) that were almost absent in those treated with unspecific intrabodies (Fig. 4a, panels R4sec, R4nuc).

Apoptosis and necrosis are the two major cell death pathways, and can be distinguished based on morphological and molecular criteria [24]. To verify which of these two processes takes place following treatment with intrabodies in vivo, an immunohistochemical staining protocol was applied to discriminate between apoptotic and necroptotic cell death through a single staining procedure on tissue sections of TC-1 tumors treated with scFvI7nuc, scFv43M2SD or scFvR4nuc and scFvR4sec as described in Materials and Methods.

As shown in Fig. 4b, only apoptotic death was revealed in the tumors treated with the anti-E7, or anti-E6 intrabodies (panels 43M2SD and I7nuc), while rare apoptotic areas were detected in tumors treated with unspecific intrabodies (panels R4sec and R4nuc). Both pathologists scored the same percentage of apoptotic areas, corresponding to 60% for scFv43M2SD and 30% for scFvI7nuc. A percentage of 5 and 15% was detected in tumors treated with the R4sec and R4nuc irrelevant intrabodies, respectively.

## Discussion

HR HPVs, together with environmental and genetic cofactors, can cause cancer in different body districts. The E6 and E7 oncoproteins of HR HPVs play a key role in cellular transformation and maintenance of the transformed status. This circumstance and the unique localization of E6/E7 within tumors can guide therapeutic approaches that are safe and precise because they target such oncoproteins.

The approach we used relies on specific scFvs characterized in previous studies [14–16] .Confocal microscopy results suggested that a key mechanism underlying the antitumor activity of both the anti-16E6 scFvI7nuc targeting cell nucleus and the anti-16E7 scFv43M2SD targeting ER intrabodies is the delocalization of oncoproteins. Furthermore, through immunological assays and Surface Plasmon Resonance (SPR), we showed that the activity of the anti-16E7 scFv43M2SD depends to some extent on interference with the E7/pRb binding [18], whereas the anti-16E6 scFvI7nuc activity seems to be at least partly related to the p53 rescue, with consequent increase of cell death due to necrosis and apoptosis [16]. Interestingly, a p53 rescue suggestive of cross-reaction was observed after transfection with scFvI7nuc of Me180 cell lines, which harbor the HR HPV68 and express an E6 protein with homology of 61.43% to the 16E6, and not after transfection of HeLa cells, harboring the HPV18 and expressing an E6 protein with homology of 57.97% to the 16E6 [25, 26]. This observation suggests the advisability of testing possibly available scFvs, against more than one related HPV genotype, to investigate potential broader activity spectra.

Recently, we also compared our scFvs to Clinical-Stage Therapeutic antibodies (CSTs) by computational analysis, and observed that they have properties well-conforming antibodies which have already reached Phase I clinical trial [27].

Delivering proteins to the cytosol is challenging and the same is true also in the case of scFvs. In vivo gene therapy using DNA delivery is a well-established procedure, with clinical trials in progress and some already available drugs such as the ZOLGENSMA® (onasemnogene abeparvovec-xioi), recently approved by FDA for muscular spinal atrophy. In this study, intrabodies were expressed directly in the target tumor cells by electroporating tumors with scFv-expressing recombinant plasmids. However, direct delivery of scFvs as proteins is also feasible [28].

The main goal of this study was to validate the antitumor activity of the previously characterized scFvI7nuc and scFv43M2SD in animal preclinical models that mimic a condition as close as possible to that occurring in humans, where a tumor lesion is already established at the time of diagnosis. The ability of counteracting tumor growth by intratumor expression of the scFvs was investigated in different mouse models.

In the first HPV tumor model used, the TC-1-LUC cells, which allow monitoring of tumor growth in tumor-bearing mice through luminescence imaging, were employed [29]. By luciferase measurements, we could visualize a decreased luciferase activity related to both the scFvI7nuc and scFv43M2SD expression, not observed in cells expressing the scFvR4nuc and scFvR4sec used as negative controls.

A critical situation of more advanced tumor progression was then simulated by using a fourfold higher number of normal TC-1 cells to implant HPV tumor in syngeneic C57/BL6 mice. Tumor growth inhibition was obtained even under these more aggressive experimental conditions.

To ensure that the scFv therapeutic efficacy did not depend on the animal model, the effect of scFvI7nuc was evaluated in a mouse model based on C3 cells, which express slightly higher levels of E6 and E7 with regard to TC-1 cells [30]. The results were comparable to those obtained with TC1 cells. It is to note that all the treatments have been started on well-established tumors while many studies reporting more effective immunotherapies on similar models have been conducted at an early stage of tumor development [31]. Interestingly, the level of tumor inhibition in the TC-1 model was similar to that obtained by inducing cell apoptosis with different treatments [32].

The incomplete inhibition of tumor growth stresses the importance of dosage and timing for efficacy of the scFv treatment, and does not exclude that increased doses of scFvs or a different treatment schedule might result in complete tumor inhibition. Of note, the scFv delivery system based on the electroporation of recombinant plasmids has high safety features that make it suitable for use in humans as well. However, different delivery systems may even increase efficacy of the treatment with scFvs. In this perspective, we are exploring an exosomes-based delivery system which has the capacity of auto-implementing [33].

Histological observation of tumors treated with therapeutic scFvs highlighted the presence of large areas of necrosis that could be due to scFv-induced apoptosis and were almost absent in tumors treated with the irrelevant scFvs. Staining of tumor sections for active caspase-3, which is a major player in the apoptotic process, revealed a high percentage of caspase-3 positive areas in tumors treated with therapeutic scFvs compared to controls. This finding indicates a direct involvement of the scFvs in the apoptotic process thus suggesting a possible biological reason for their therapeutic effect.

The apoptotic process induced in mice tumors by scFv treatment is in agreement with data previously obtained in vitro in HPV16-positive cell lines, where we demonstrated the scFvI7nuc involvement in hampering E6-dependent p53 degradation and rescuing pro-apoptotic activity of the tumor suppressor [16]. The results presented here conclusively demonstrate that both treatments with anti-16E6 or -16E7 intrabodies induce strong apoptosis in tumors.

As far as concerns the scFv43M2SD action, we believe that it may have effects in more than one direction. We have already demonstrated that the scFv43M2SD binding to E7 interferes with the pRb binding and degradation, thus increasing the pRb intracellular pool and reestablishing anti-proliferative activity of the tumor suppressor [15, 18]. However, non-nuclear activity of pRb was reported in the induction of mitochondrial apoptosis via direct interaction of pRB with Bax [34]. Accordingly, a fraction of endogenous pRB is constitutively associated with mitochondria. Hence, it could be hypothesized that the decreased pRb degradation in 43M2SD-expressing cells may increase mitochondrial pRb levels thereby inducing the intrinsic pathway of apoptosis.

Nevertheless, it cannot be excluded that scFv43M2SD, in virtue of the SEKDEL signal, may exert anticancer activity also through a mechanism involving the ER stress. Indeed, over-expression of proteins binding to the SEKDEL receptors on ER was shown to induce the release of important mediators of ER homeostasis and the ER stress response [35, 36]. Both the ER stress response and the activation of the mithocondrial pathway of apoptosis may contribute to the tumor growth inhibition after the scFv43M2SD delivery to tumor cells.

**Fig. 5:**
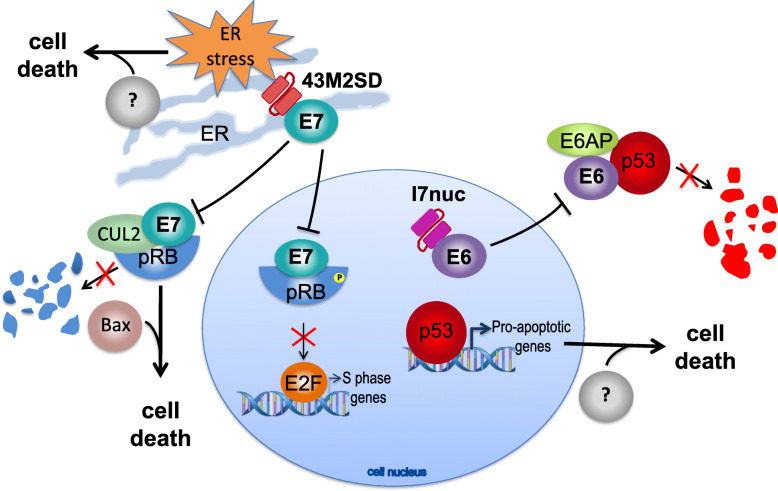
Hypothetical mechanisms of induction of apoptosis by the anti-16E6 and anti-16E7 intrabodies. The binding of scFvI7nuc (I7nuc) to E6 can inhibit the cytoplasmic degradation of p53. The restored levels of nuclear p53 may activate the transcription of pro-apoptotic genes including Puma, Noxa, Bak and Bax and the subsequent loss of mitochondrial membrane potential, with downstream activation of the executor caspase 3, finally leading to cell death. The binding of scFv43M2SD (43M2SD) to E7 can inhibit its translocation to nucleus and the subsequent inactivation of the Retinoblastoma tumor suppressor (pRB) thus restoring the control of E2F transcription factors by pRb. At the same time, the binding of scFv43M2SD can inhibit the association of E7 with the cullin 2 complex (CUL2) and the recruitment of pRB for ubiquitination. Increased levels of pRB, regardless of its role as a transcriptional regulator, can directly activate the BAX apoptosis regulator at mitochondrial level and promote cell death [34]. The binding of scFv43M2SD to KDEL receptors of ER may also induce ER stress-related molecules which can cause apoptosis by triggering the activation of caspases

In Fig. 5, a schematic representation of the hypothetical and demonstrated effects of intrabodies targeting the oncoproteins, is shown.

Although the E6 and E7 action has been extensively studied, it should not be excluded that in the future new functions performed in other cellular compartments will be discovered. In this regard, the implementation of new constructs to provide these or other scFvs with different signals for intracellular localization may represent a useful opportunity. Different antigen-specific scFvs can bind to different epitopes of the same protein thus potentially interfering with different protein functions. This may have therapeutic relevance since the pro-tumor action is a mosaic of activities that could be prevented as a whole or even modulated separately. Furthermore, since the two oncoproteins exert a concerted action, the concomitant inhibition of E6 and E7 may be more efficacious than the inhibition of only one of them; for this reason, it could be useful to study the effect of anti-16E6 and anti-16E7 scFvs in combination.

## Conclusion

This study shows in preclinical models the efficacy of two intrabodies against the E6 and E7 oncoproteins in counteracting the growth of HPV16 tumors. This represents a step forward for the treatment of HPV tumors, but further studies will be needed to optimize delivery, doses, timing and number of administrations for their translation to the clinic. The localized expression of the transgene increases the safety of the treatment, which can be repeated as many times as necessary since the plasmid vectors are free from undesired pathogenicity and immunogenicity. Furthermore, the effectiveness of electroporation, a methodology allowed for human treatment, to deliver scFvs to tumors was confirmed.

## Supplementary Information

**Additional file 1: Fig. s1.** Antitumor effect of scFvs delivered to TC-1-luc HPV tumors. Imaging of single mice challenged with TC-1-luc tumors and treated with scFvI7nuc, scFv43M2SD or scFvR4nuc and R4sec as controls. Treatments were delivered four times at one-week intervals (from T1 to T4). Mice are indicated by numbers on the side. T is the time in weeks after tumor cell challenge. Luminescence was quantified as described in Methods at indicated time points before the sacrifice of mice for ethical reasons (†).

## Data Availability

The data that support the findings of this study are available from the corresponding author, upon reasonable request.

## Abbreviations

HPV: Human Papillomavirus
HR: High Risk
SCC: Squamous Cell Carcinoma
HNSCC: Head and Neck Squamous Cell Carcinoma
TAA: Tumor Associated Antigen
CTL: Cytotoxic T-Lymphocyte
mAbs: Monoclonal Antibodies
VH: Variable Heavy region
VL: Variable Light region
CDR: Complementarity Determining Region
ER: Endoplasmic Reticulum
NLS: Nuclear Localization Signal
s. c.: Subcutaneously
EP: Electroporation
H&E: Hematoxylin/Eosin
DAB: 3,3′-diaminobenzidine
SPR: Surface Plasmon Resonance
CSTs: Clinical Stage Therapeutics
