# Supplementary figures and images for: Intrabodies targeting human papillomavirus 16 E6 and E7 oncoproteins for therapy of established HPV-associated tumors
Source: J Exp Clin Cancer Res. 2021 Jan 23;40:37. doi: 10.1186/s13046-021-01841-w (PMC7825221; doi:10.1186/s13046-021-01841-w)

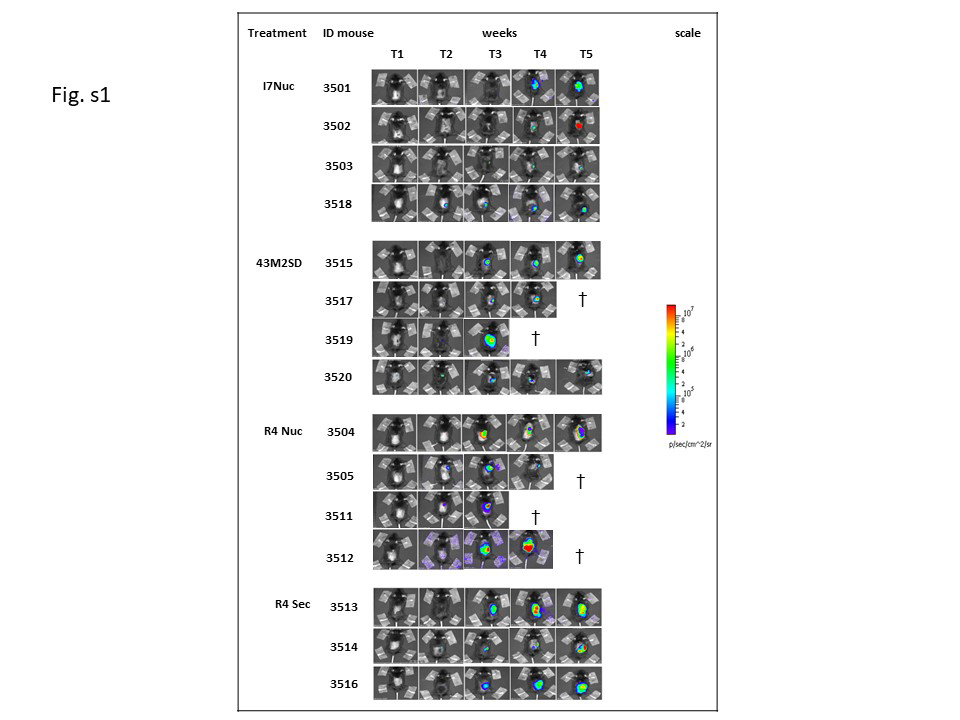

Supplement: Supplementary file 1 — Additional file 1: Fig. s1. Antitumor effect of scFvs delivered to TC-1-luc HPV tumors. Imaging of single mice challenged with TC-1-luc tumors and treated with scFvI7nuc, scFv43M2SD or scFvR4nuc and R4sec as controls. Treatments were delivered four times at one-week intervals (from T1 to T4). Mice are indicated by numbers on the side. T is the time in weeks after tumor cell challenge. Luminescence was quantified as described in Methods at indicated time points before the sacrifice of mice for ethical reasons (†). [file 13046_2021_1841_MOESM1_ESM.jpg]
